# Mining and standardizing chinese consumer health terms

**DOI:** 10.1186/s12911-018-0695-6

**Published:** 2018-12-07

**Authors:** Li Hou, Hongyu Kang, Yan Liu, Luqi Li, Jiao Li

**Affiliations:** Institute of Medical Information, Chinese Academy of Medical Sciences/Peking Union Medical College, Beijing, 100020 China

**Keywords:** Consumer health terms, Medical terms, Term mapping, Annotation, Consumer health vocabulary

## Abstract

**Background:**

Health professionals and consumers use different terms to express medical events or concerns, which makes the communication barriers between the professionals and consumers. This may lead to bias in the diagnosis or treatment due to the misunderstanding or incomplete understanding. To solve the issue, a consumer health vocabulary was developed to map the consumer-used health terms to professional-used medical terms.

**Methods:**

In this study, we extracted Chinese consumer health terms from both online health forum and patient education monographs, and manually mapped them to medical terms used by professionals (terms in medical thesauri or in medical books). To ensure the above annotation quality, we developed annotation guidelines.

**Results:**

We applied our method to extract consumer-used disease terms in endocrinology, cardiology, gastroenterology and dermatology. In this study, we identified 1349 medical mentions from 8436 questions posted in an online health forum and 1428 articles for patient education monographs. After manual annotation and review, we released 1036 Chinese consumer health terms with mapping to 480 medical terms. Four annotators worked on the manual annotation work following the Chinese consumer health term annotation guidelines. Their average inter-annotator agreement (IAA) score was 93.91% ensuring high consistency of the released terms.

**Conclusions:**

We extracted Chinese consumer health terms from online forum and patient education monographs, and mapped them to medical terms used by professionals. Manual annotation efforts have been made for term annotating and mapping. Our study may contribute to the Chinese consumer health vocabulary construction. In addition, our annotated corpus, both the contexts of consumer health terms and consumer-professional term mapping, would be a useful resource for automatic methodology development. The dataset of the Chinese consumer health terms (CHT) is publicly available at http://www.phoc.org.cn/cht/.

## Background

Internet has become a main resource for consumers to acquire health information. As reported in 2017, 54.3% of Chinese Internet users (i.e., 751 million users) sought and accessed health related information from online forum [1, 2]. Several previous studies have proved that health-related online information could affect consumers’ health-related attitudes and behaviors [3–5]. However, there is a mismatch between terms used by healthcare professionals and those used by the consumers [6, 7], it is challengeable for the consumers to accurately express their health needs using medical query terms, consumers often have difficulties in expressing and understanding the medical concepts, and consumers always use layers of language to express their health needs, almost of the online health information are organized by professional terms, thus they fail to retrieve relevant health information [8, 9]. In 2017, the Chinese citizen’s health literacy level was reported to be 12.58% according to the National Health and Family Planning Commission of China [10], meaning that only 12 individuals out of 100 are able to accurately access and understand the basic health information and services for making correct decisions through online information and services. Therefore, it is necessary to build up a bridge between the consumer and healthcare professionals.

It is also noteworthy that laypersons and healthcare experts would like to broaden the domain of health-related terms [11], for instance, “senile dementia” vs. “Alzheimer” and “甲亢(the abbreviations of Hyperthyroid in Chinese)” vs. “甲状腺功能亢进症(Hyperthyroidism)”, or“心梗”(the abbreviations of myocardial infarction in Chinese)vs. “心肌梗塞”(myocardial infarction). Professionals or experienced patients have greatly provided online health information in which a mismatch in the terminology and style of writing attenuates laypersons’ ability. On the other hand, professional needs and scientific discoveries were supported as a precious source of information leading to extensively utilization of online forum data. For instance, search queries, made by millions of users throughout the world, were scrutinized by experts and scholars in Google Company to identify the outbreak and spread of influenza-like epidemics with a relatively acceptable accuracy [12]. Therefore, text corpora from online question-answer platform must be a proper corpora to find the health-related concepts expressed by laypersons.

Many professional vocabularies have described and classified medical concepts, such as the Systematized Nomenclature of Medicine-Clinical Terms (SNOMED-CT) [13], National Cancer institute thesaurus (NCIt) [14], International Classification of Diseases 10, 10th Edition (ICD-10) [15], and other biomedical vocabularies or ontologies included in the Unified Medical Language System (UMLS) Metathesaurus, while vocabularies reflecting consumer-oriented health terms significantly suffer from being mature. Zeng and Tse explored and developed Consumer Health Vocabularies (CHV) to narrow the gap between the laypersons and health professionals in the USA [16, 17]. Several studies have conducted automatic term identification for developing the CHV [18, 19]. N-Gram and manual review were the major methods to extract the candidate terms. Zeng et al. [20, 21] used queries put to a Find-A-Doctor site and MedlinePlus, they found problems at the lexical level (e.g., spelling, morphology, and word order) and at the semantic level (e.g., synonymy). Smith et al. [22] used emails submitted by consumers to a cancer information service. They extracted 504 unique terms representing “features and findings,” and mapped them to the UMLS. For truly perceiving the CHV, two survey instruments were developed for assessing surface-level familiarity and concept-level familiarity of CHV term [23]. It showed that the development of consumer health vocabularies for English is relatively mature. In China, Chinese Medical Subject Headings (CMeSH) is a kind of professional vocabulary, which is widely used in annotating Chinese medical literature [24], and also applied to recognize the medical concepts in the Chinese medical text [25], most of the terms in CMeSH are very obscure for consumers to truly understand, while there is no CHV in China.

In the clinical domain, various natural language processing(NLP) systems for Chinese clinical text have been created, such as named entity recognition [24], clinical information extraction [26, 27], and speculation detection [28]. The main challenges in these tasks include word segmentation and feature representation and selection. To our knowledge, there were only few studies that have investigated CHVs used by consumers and standardized in Chinese. In order to enhance the consumer’s ability in finding and understanding health information, it is necessary and urgent to extract relevant medical terms from community-generated data and monographs of patient’s education, as well as mapping a relationship between the consumers’ expression and professionals’ expression on health information.

## Methods

**Fig. 1:**
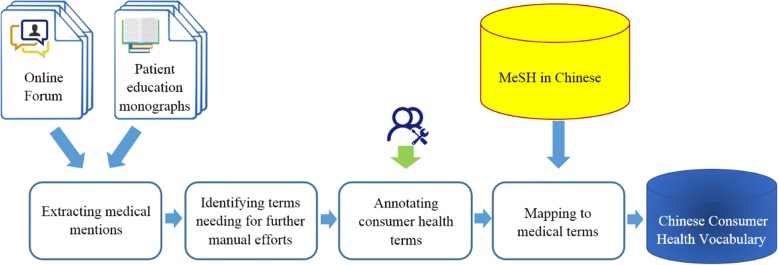
Workflow to mine and standardize consumer health terms

We presented the process of identification and extraction of consumer’s medical entities and their alternative synonyms from community-generated data and monographs of patient’s education by manual annotation approach. Figure 1 displays the workflow, and the following paragraphs describe the process as well.

### Collecting consumer health corpus extracted from online forum and patient education monographs

Text corpora from online forum about health was served as a valuable resource to extract laypersons’ expressions of medical terms and their corresponding professional expressions. Consumers can easily find the correct answers about the treatment of a particular disease, side effects of some medications, symptoms of different illnesses from online forum about health, such as Baidu Knows, which is a Chinese language collaborative Web-based collective intelligence by question and answer sharing platform with more than 100 million users, and which was provided by the Chinese search engine Baidu [29].

A series of articles are questions posted by health consumers from the Health and Medicine section on this online forum [30]. There are more than 30 sub-categories in the Health and Medicine section. For example, Pediatrics is a sub-category of Health and Medicine, and Department of Gastroenterology is a sub-category of Medicine. The complete category hierarchy can be repeatedly obtained by traversing the sub-category links.

We parsed the web page, including all categories related to clinical departments in the website, extracted the web page’s feature, used MyEclipse as a developing tool and MySQL as a data storage tool, and employed HtmlCleaner which is an open-source HTML parser written in Java. Then, we selected the time interval from Sept. 2017 to Feb. 2018. Finally, we received 1,414,327 questions through this website.

Another material was patient’s education monograph that contain 1428 articles about the prevention and treatment of chronic diseases, frequently-occurring diseases, and drug safety [31], which were written by a lot of popular science experts who aimed to disseminate popular science knowledge in medical fields to the public. In these monographs, each knowledge article began with one question and one answer, and then continued with several passages. All the knowledge in this monographs was expressed in popular science language to popularize the professional knowledge, and therefore, we selected these monographs as the corpora to extract names of the medical terms and their alternative synonyms.

### Extracting medical terms and their common alternative synonyms

We performed manual annotation to extract disease mentions from the questions of online forum and patient’s education monographs. This procedure was initiated with data processing to filter the same professional terms as those in Chinese Medical Subject Headings (CMeSH) before an annotator conducted extraction. To ensure the accuracy of extracted medical terms and their common alternative synonyms, we used 3 groups of CMeSH annotators to extract the medical terms from these corpus, and each group had two indexers, where one indexer group annotated the corpus from online forum, the another indexer group annotated the corpus from monographs, and the third group was responsible for verification.

#### Data preprocessing

We designed different methods to preprocess the data extracted from different sources. For data extracted from online forum, we designed a filtering mechanism to pre-process the raw data and improve the efficiency, as the number of questions extracted from online forum were up to 2,000,000. For data achieved from monographs, there were some general characteristics in sentences, and thus, we can automatically process the part of materials that relied on computer systems. The preprocessing workflow was presented as follows:

##### Filtering the professional terms that are as same as in CMeSH

Firstly, we processed an exact match algorithm with the terms and entry terms in CMeSH, if a medical term in a question was same as CMeSH, we filtered the sentence, and the rest of materials were about 700,000 messages after the experiment. Secondly, 8436 questions from four departments, including department of endocrinology, department of cardiology, department of gastroenterology, and department of dermatology were randomly selected as the sample for annotated corpus.

##### Identifying medical terms and their synonyms from monographs with text-mining

Layperson can easily understand the patient education monograph. Firstly, we selected the articles related to the four departments of endocrinology, cardiology, gastroenterology and dermatology, and there were 345 articles related to these four departments in the monographs after manually reviewed by the annotator. Many definitions and introductions were written with alternative forms of disease, such as abbreviations or alternative names which that are following or adjacent to the first occurrence of this disease, we call these words as “AKA(also known as)” terms. For example, one title of the article was “What is cold infection? [32]”, which started as: “Cold is also called as Common Cold and Upper Respiratory Infection……” informing the readers that “Common Cold” and “Upper Respiratory Infection” were the alternative names for “Cold”(Table 1 Example 1). The AKA terms provide the feasibility for making automatic text mining and information extraction, and we thus identified a list of phrases that generally connected a disease concept term to its alternative terminology. In this study, we have 18 AKA terms, such as “简称”,“俗称”, and “又称”, etc. (Table 2). More illustration about AKA terms and the mechanism of automated text-mining synonyms were shown in Table 1. We identified 120 synonyms pairs from 345 articles by automatic term identification and synonym detection, after reviewed by annotators, the score of precision was 100%.

**Table 1:** AKA terms and examples in Patient education monographs

T_1_ [AKA] T_2_, T_3_, …	
Example 1:	
Cold is also called as Common cold and Upper Respiratory Infection T_1_: Cold T_2_: Common cold T_3_: Upper Respiratory Infection [AKA]: is also called as	
Example 2:	
Influenza is called flu for short T_1_: Influenza T_2_: flu [AKA]: is called	
Example 3:	
Hyperthyroidism is referred to as “Hyperthyroid” T_1_: Hyperthyroidism T_2_: Hyperthyroid [AKA]: is referred to as ………

**Table 2:** Patterns of detecting synonyms of health terms

Patterns in Chinese	Patterns in English	Patterns in Chinese	Patterns in English
A简称B	A be called for short B	A俗称B	A commonly called B
A简称为B	A for short B	A全称是B	A full name B
A中文简称B	A shortened form in Chinese B	A全称为B	A full name is B
A缩写B	A abbreviation B	A又名B	A also called B
A又称B	A also called B	A通俗的讲B	A commonly known as B
A又称为B	A also called B	A又叫B	A also referred to as B
A亦称B	A also called is B	A也称B	A also referred to as B
A亦叫B	A also named B	A也称为B	A also referred to as B
A亦作B	A sometimes called also termed B	A通俗称谓是B	A commonly called name B

#### Annotating health terms by a manual approach

After two automated data processing stages, we recruited CMeSH indexers to annotate terms from two types of corpus, in which the Annotators’ work patterns and Annotation guidelines were illustrated in detail.

##### Annotators

For entity annotation, we recruited four CMeSH indexers in two groups, and all of them had a medical training background and curation experience. One group was responsible for query data, and another group was responsible for plotting monographs. Each query sentence was independently annotated by two annotators (i.e., double-annotation). Differences were resolved by a third and a senior annotator.

##### Annotation guidelines

In this task, we annotated only the disease name and their alternative names used or perceived by consumers. To ensure the scientificity and conformity, the CMeSH annotators were asked to follow a set of guidelines when annotating the questions from online forum and the sentences from monographs: Firstly, if there were two or more medical disease terms in one sentence, they should annotate all the terms, and separated these terms by a comma in one row of a table. Secondly, for questions from online forum, the annotators should extract the terms expressed by layperson fully based on the original, and they could not use their professional knowledge to exchange the consumers’ expression to professional one, including the abbreviation of diseases, even wrong spelling used by consumers. For example, one question was “高血压性的肾病怎么办?(How do I do if I have hypertensive nephrosis?)”, the disease term extracted from this sentence should be “高血压性的肾病 (hypertensive nephrosis)”, it should not be annotated as “肾性高血压 (Renal Hypertension)”. For monograph, regardless of whether the term was the same as the term in the CMeSH, the annotators need to extract the terms expressed as they were in books, and reviewed the data processed during the preprocessing. Thirdly, after finishing all the consumers’ expression about disease, the annotators were asked to annotate the CMeSH terms against the consumers’ term annotated, if there was not exactly equal CMeSH terms, the closest terms or its father node in CMeSH should be selected. For example, the consumer health term “克山病(KeShan disease)” is a kind of myocardiopathy that is prevalent in KeShan areas of China, and there is no exactly equal CMeSH term. So, the annotators should select the closest term “Myocardiopathy”, and we could generate the conceptual pairs, each pair of disease concepts including one professional term and more consumer alternative names through this annotation. Eventually, discrepancies and questions on annotations were discussed and settled by a senior annotator. The annotation examples are presented in Table 3.

**Table 3:** Annotation examples

Query sentences	Consumer expression	CMeSH term
为什么人会有三高? (Why Does people have “Three High”?)	三高 (Three High)	高血压、高血糖、高脂血症 (High blood pressure 、Hyperglycemia、Hyperlipidemias)
甲亢病人能吃安眠药吗? (Can patients with hyperthyroidism eat sleeping pills?)	甲亢 (Abbreviation of Myocardial Infarction in Chinese)	甲状腺功能亢进症 (Hyperthyroidism)
三高人能吃猪肝吗? (Can patients who have “Three High” eat pork liver?)	三高 (Three high)	高血压、高血糖、高脂血症 (High blood pressure 、Hyperglycemia、Hyperlipidemias
党参能治心梗吗? (Can Codonopsis cure myocardial infarction?)	心梗 (Abbreviation of Myocardial Infarction in Chinese)	心肌梗死 (Myocardial Infarction)
鹿心对心衰是否有效 (Does the deer heart is effective for heart failure?)	心衰 (Abbreviation of Heart Failure in Chinese)	心力衰竭 (Heart Failure)
高血脂的患者吃什么东西好? (What’s good for the patients with high blood lipids?)	高血脂 (High blood lipids)	高脂血症 (Hyperlipidemias)

##### Mapping CMeSH term pairs to MeSH ID

Since CMeSH is the Chinese version of MeSH, each medical subject heading in CMeSH corresponds to that subject heading in MeSH, CMeSH terms can be automatically connected to MeSH ID through data processing. After the manual annotated, we got too much terms pairs, we mapping CMeSH terms pairs to the MeSH ID according to the CMeSH terms, if there were two consumer health terms mapping to one same CMeSH term, then the two consumer health terms have the same MeSH ID. For example, we annotated the subject heading terms in CMeSH for “高血压病”,“原发性高血压”as “高血压(hypertension)”, then we would give the MeSH ID as “D006973”for the two consumer health terms “高血压病” and“原发性高血压”.

#### Data storage methods

In order to storage the health terms used by consumers and its equal professional consumers, and make the pairs of concepts to be easily browsed for study, we represented disease terms according to the method of concept organization in UMLS [33]. As shown in the following, we attempted to design a data management and storage method for disease terms based on the framework of concept organization, and defined the naming rules by describing different entities of diseases, and defined every element in the Concept Table, including the meaning of elements and elements’ abbreviation. The elements that referred to were defined as the unique identifier, source, string, English name, note, tree number, MeSH ID, frequency, etc. In order to achieve the frequency of each extracted disease name, we counted the frequency of each annotated disease name with the help of an exact match algorithm, Table 4 showed the knowledge representation examples of consumer health terms.

**Table 4:** Data storage examples of consumer health terms

CVc-CUI	String	Name_En	SAB	MeSH ID	Department	FREQ
C00104	甲亢	Hyperthyroidism	Online Forum	D006980	Endocrinology	18,231
C00116	中风	Stroke	Online Forum	D020521	Cardiology	8553
C00319	拉肚子	Diarrhea	Online Forum	D003967	Gastroenterology	7719

CVc-CUI: Unique identifier for health term, STR: String of consumer health term, Name_En: English String of the term, SAB: Abbreviated source name, MeSH ID: Unique ID of MeSH, FREQ: Query frequency of terms

### Inter-annotator Agreement (IAA) analysis

To assess the consistency of the disease and chemical annotation, we measured pairwise agreement for duplicate annotations using Jaccard Score [34]. As shown below, if we defined A as the set of mentions of team A, B as the set of mentions of team B, then the Jaccard’s score, *S*_*A*, *B*_ could be obtained by counting the number of agreements and disagreements. Mentions with the same ID, start and end point and concept identifier were counted as a case of agreement. For example, if one annotator annotated “renal hypertension” with MeSH ID of D006978, another annotated “renal hypertension” with MeSH ID of D006977, then that would count as two cases of disagreement and no case of agreement as different mentions were annotated.

$$ {S}_{A,B}=\frac{\mid A\cap B\mid }{\mid A\cup B\mid } $$

## Results

### Dataset overview

The corpus was consisted of two sets of materials with diseases. The materials obtained from the online forum were produced by layperson, and the other materials were derived from patient education monographs, and were written by experts who could popularize professional terms. We identified 1349 disease mentions from 8781 articles in online forum and monographs, and after filtering the same terms as the term in the CMeSH by manual annotation, the number of consumer disease terms was 1036, showing that some consumers have higher health literacy, and many terms of common diseases are queried repeatedly. From the dataset, we can know that consumer health terms are the abbreviations of the professional terms in informal expression, or are the general expression of the medical terms, or are the typos in Chinese but which are very familiar by consumers. Most of consumers always use the typos to retrieve health information in online forum, such as the term “埂” in “脑埂” (the abbreviation of “Brain Infarction”), “埂” is a typos of “梗”, but most consumer always use “脑埂” to express the professional terms “脑梗死(Brain Infarction)”. As shown in Table 5, the two datasets have different distributions of disease mentions, making the dataset more useful for training the models. The table also showed that there were more disease mentions and disease entities (IDs) in patient education monographs than disease mentions and disease entities (IDs) in the online forum, while the number of corpora from online forum was larger than the number of corpora from monographs, illustrating that the disease is the subject of internet for consumers, and that is relatively focused on few medical departments, and there were numerous same questions with the same disease terms. However, patient education monographs aimed to explain the professional knowledge, and there were many definitions, and so we could extract more disease mentions by monographs.

**Table 5:** The overall of consumer health term datasets (CHT)

Resource	# of Articles	# of All Terms	# of Consumer Health Terms	# of Medical Terms (MeSH_IDs)
Online Forum	8436	628	479	243
Monographs	345	721	577	301
Total	8781	1349	1036	480

### Inter-annotator agreement for mention annotation

The IAA scores from forum and monographs were 92.5 and 95.3%, respectively. The IAA scores over the entire corpus were 93.91% (diseases), demonstrating higher agreement on monographs than online forum. By analyzing the disagreements, we found that most of them were associated with the uncertainty of the disease terms expressed by consumers. For example, in one query sentence entitled “吃新鲜黄秋葵降三高吗?(Can eat okra reduce three high?)” , “三高” in Chinese language is the abbreviation of high blood pressure, high blood sugar, and high cholesterol, and it was difficult to indicate whether to annotate “three high” as “high blood pressure” (MeSH ID: D006973) or annotate “Hyperlipidemias” (MeSH ID: D003872) or “Hyperglycemia” (MeSH ID: D006943). Eventually, there were many cases of disagreement over the concept identifier of diseases, especially for the mentions where the text did not exactly match any CMeSH term. In a number of cases, it was difficult to determine whether to assign an unknown concept identifier of “-1” or an ancestor concept identifier.

### Dataset access

**Fig. 2:**
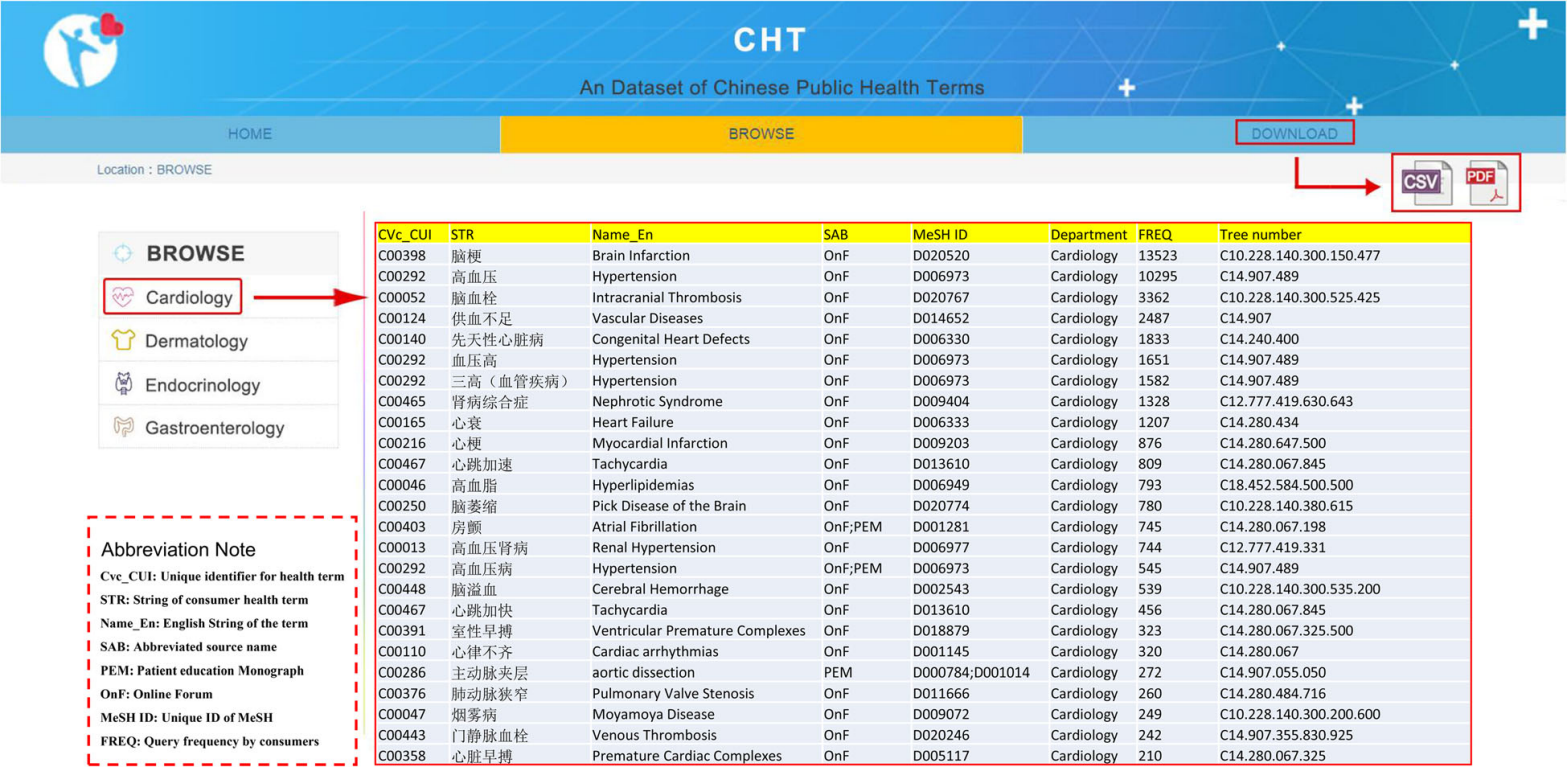
User interface of CHT Dataset

We provided a user-friendly interface that enabled users to access the CHT dataset (Fig. 2). In the “Home” page, users can know about the basic information and the purpose to build this dataset. In the “Browse” page, users can obtain the detailed information of health terms including the string of the term, its English name, MeSH ID, its popularity degree in Chinese consumers, its tree number in MeSH, etc. Users can also browse the terms by different medical departments. In the “Download” page, users can download all the standardized Chinese public health terms in CSV, and PDF formats to use them conveniently according to their usage purposes.

## Discussion

### Query frequency of disease mention from different sources

**Fig. 3:**
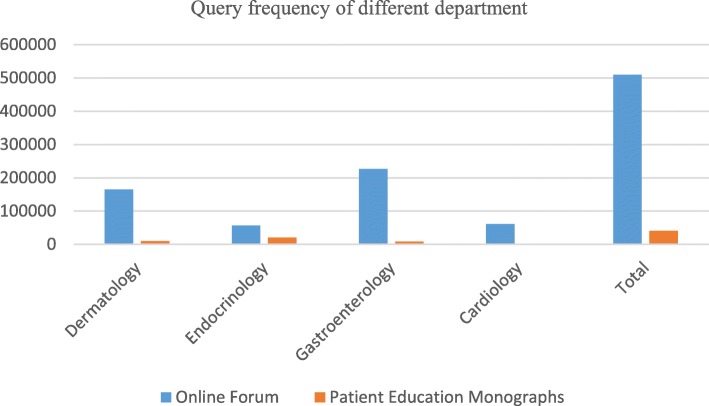
Query frequency of different medical departments in different sources

By accurately matching the extracted terms with the initial data collected from online forum, we counted the query frequency of each extracted disease term, and calculated the frequency of each term from forum as well as monographs. After that, we obtained the query frequency of four medical departments from different sources. The proposed approach for manually extracted health terms by consumers showed that the terms extracted from online forum had higher familiarity than those terms extracted from patient education monographs. Figure 3 shows a great distinction in different sources. The questions from online forum are posted by consumers, and the terms are expressed by consumers. Therefore, the frequency of terms from online forum is higher than that of the terms from the monographs. The data from patient education monographs is in line with consumers’ expression habits to some extent, which testifies that the professional and consumer understand and express the medical terms in different ways. From the distribution of the query frequency in different medical departments, it is demonstrated that gastroenterology and dermatology have the highest frequency in Chinese public, for example, “Liver Cancer”and“Gastric Cancer” have the highest frequency terms for consumers inquiries. All the CHT datasets are freely available and can be downloaded by the research community members through the website link, http://www.phoc.org.cn/cht/.

### Disease mention distribution in four departments

**Fig. 4:**
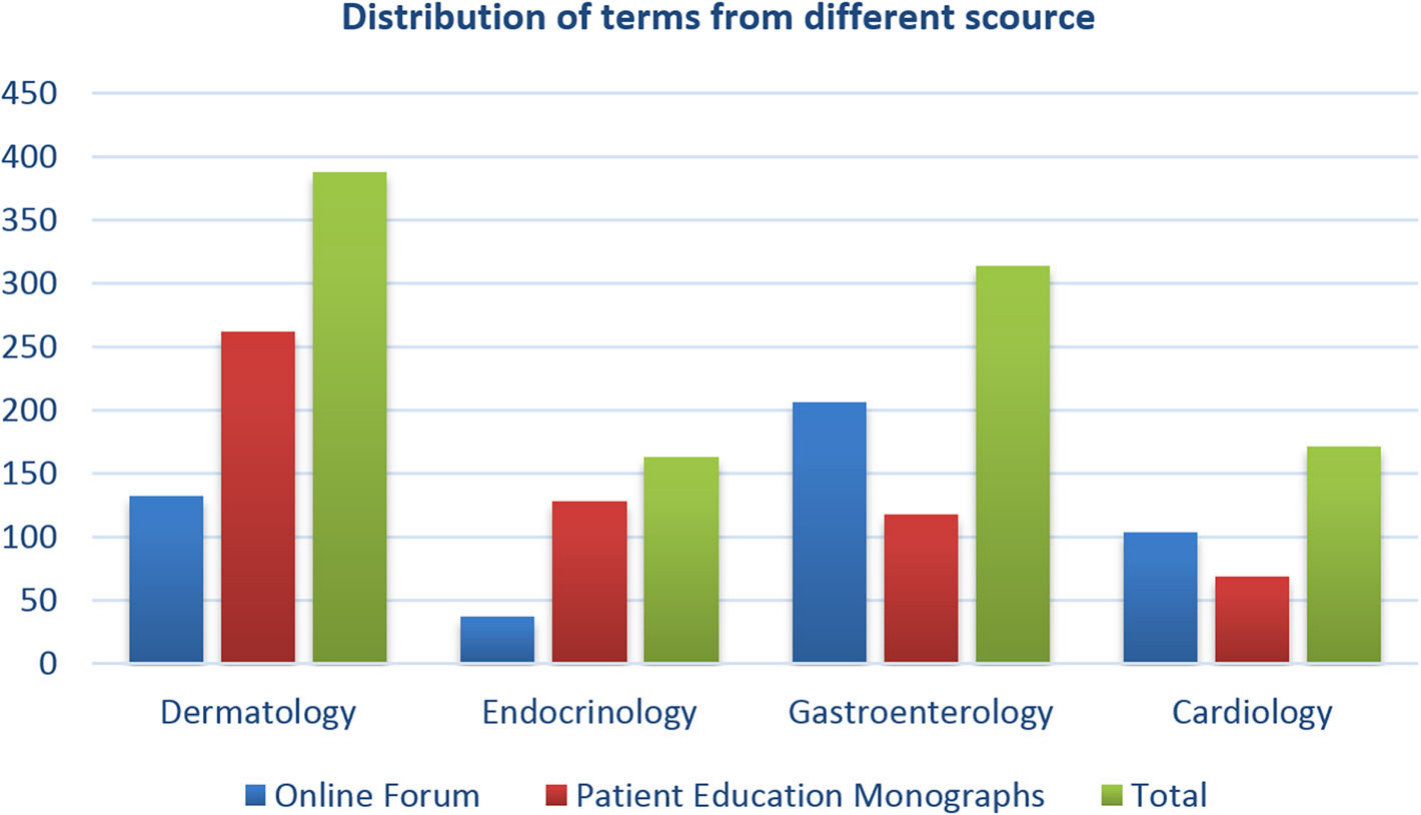
Distribution of disease terms in different sources. The number of disease terms extracted from Online Forum in the departments of dermatology, endocrinology, gastroenterology, and cardiology is 132, 37, 206, and 104, and the number of terms from monographs in the four departments is 262, 128, 118 and 69. The total number of disease terms in the four departments is 388, 163, 314, and 171

**Fig. 5:**
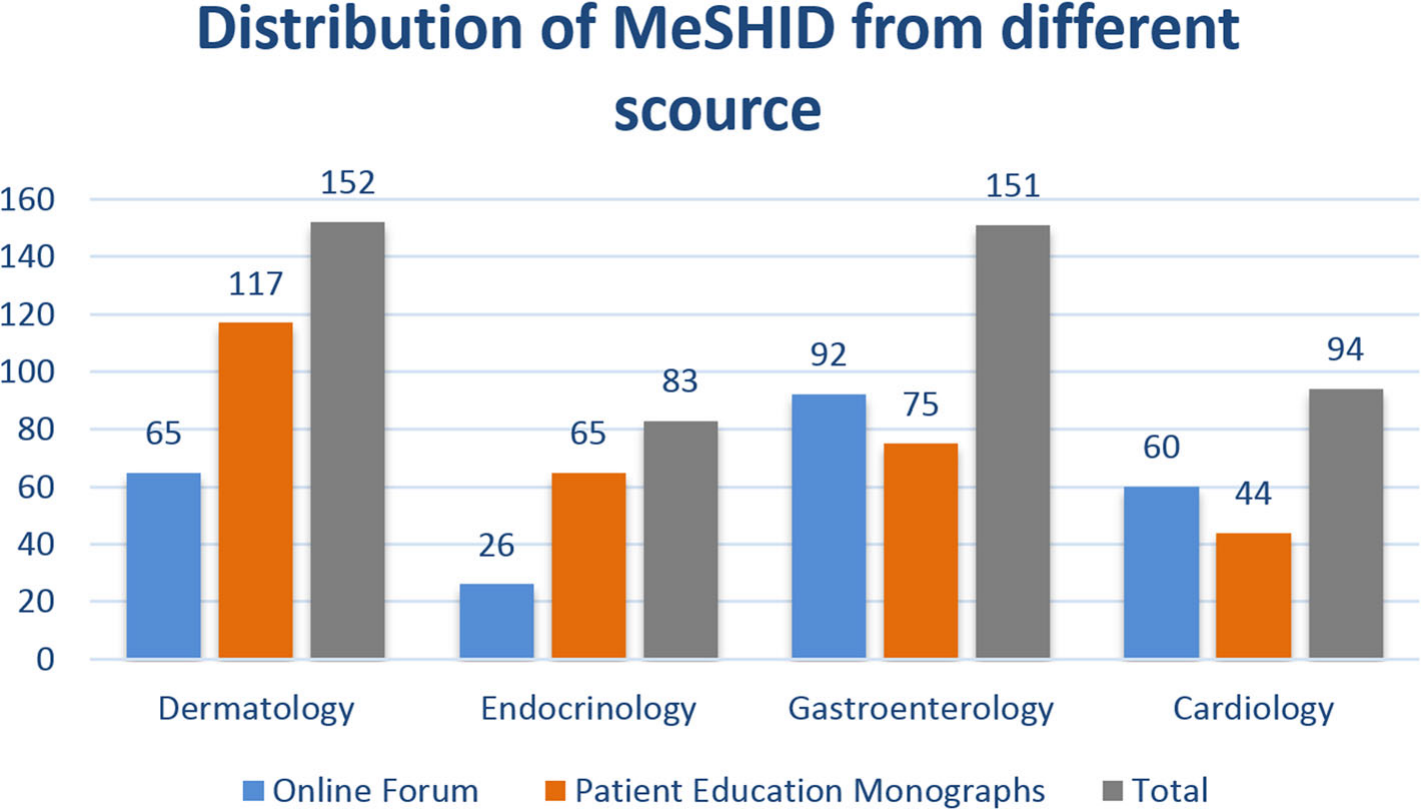
MeSH ID distribution in different sources. The number of diseases MeSH ID extracted from Online Forum in the departments of dermatology, endocrinology, gastroenterology, cardiology is 65, 26, 92 and 60, and the terms extracted from monographs in the four departments is 117, 65, 75 and 44. The total number of disease MeSH ID extracted from the four departments is 152, 83, 151, and 94

The number of diseases annotated from different sources is not the same, and they can complement each other. Figures 4 and 5 shows the distribution of diseases in different sources. On an average, the disease terms from online forum are less than those from patient education monographs, there are more terms from online forum than from monographs in department of gastroenterology, and there are more terms from monographs than from online forum in department of dermatology. This shows that compared with the dermatological diseases terms, the public is more familiar with the terms of gastroenterology and cardiology, and there are more terms that can be supplemented to consumer health terms from monographs in the department of dermatology. So are the same findings with regards to the distribution of MeSH ID. In the same time, it can be shown that most of the terms extracted from the forum and monographs are different, and these type of data sources complement to each other, which subsequently helps us to build a more perfect consumer health vocabulary.

### Overlap of disease mention among different sources

**Fig. 6:**
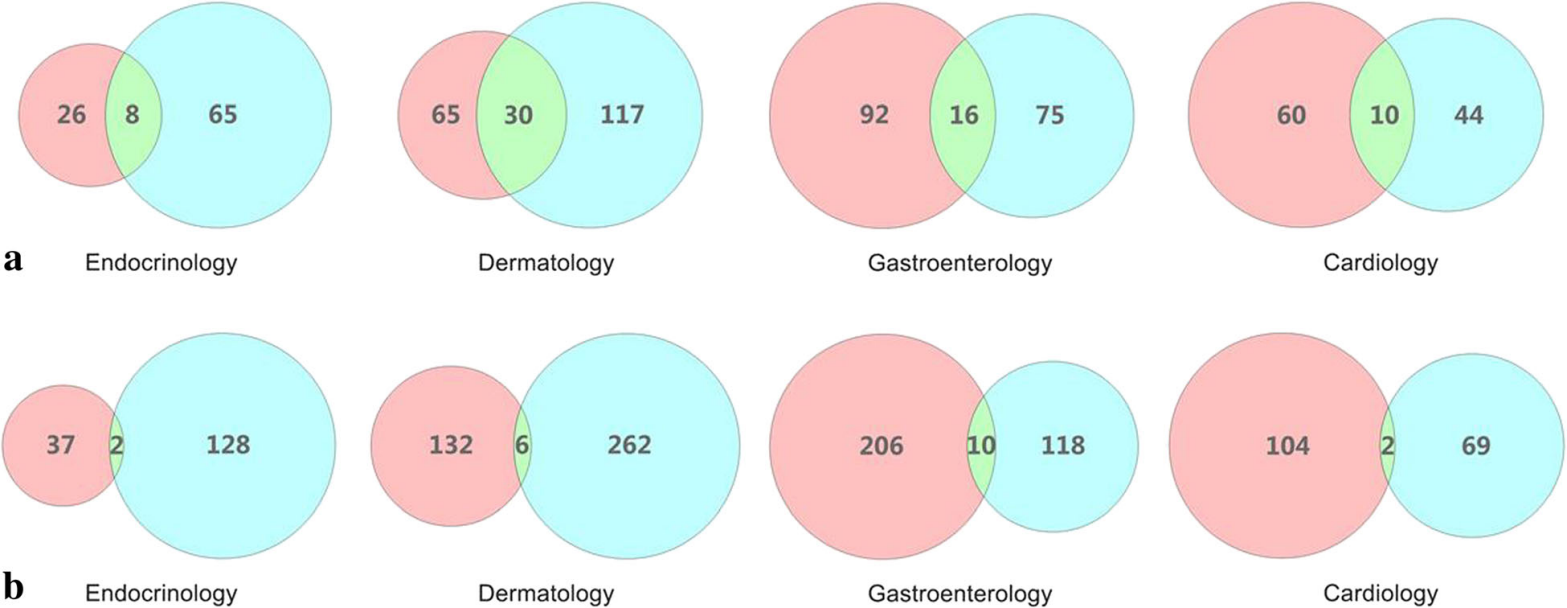
Overlap of disease mention from forum & monograph. **a** Overlap of disease MeSH ID extracted from forum & monograp. **b** Overlap of disease term extracted from forum & monograph. The pink circle is the number of terms from forum, the blue circle is the number of terms from monograph, and the green circle is the overlap of terms from forum & monograph

Although the corpus can complement each other, there is some overlap in the disease terms from different sources. Figure 6 shows the overlap of disease entity from four departments, including terms and MeSH ID extracted from different corpora. This demonstrates that the MeSH ID is more same in the departments of dermatology and gastroenterology, indicating that one MeSH Heading term has many synonyms in these departments, and there are differences in the expressions between the terms used by consumers and those written by experts. This suggests that consumers and patient education experts have higher inconsistencies in terms of expression in the department of dermatology. On the other side, it indicates that the overlap of unique disease mentions is less in the four medical departments, and also suggested that the consumers have more informal expressions on disease terms, proving that these two types of sources are good supplements of corpus to extract the consumer health terms.

### Limitations and future studies

Extraction of Chinese consumer health terms was only applied in extraction of disease in this study, and the scope of category was only limited to four departments, but was far from precisely representing the consumer health vocabulary. Our next step is to mine the drugs as well as the symptom names from different sources, and the extraction area will be extended to other departments. When the dataset is sufficiently large, we will investigate the technology of named entity recognition, explore an automatically annotated technology based on the dataset of consumer health terms, and expand the scale of this dataset to support text mining. We hope that this database is mainly developed for Chinese consumer health terms and could serve as an important resource for the research and development of consumer health vocabulary.

## Conclusions

In this study, we demonstrated the effectiveness of a novel approach that used various corpora, such as questions in online forum and patient education monographs to extract the pairs of professional terms and their equivalent consumer terms, making it helpful to construct CHV corpus for text-mining in the future, and the work mapping of the consumer term to the professional terms can be helpful to reduce the gap between health professionals and laypersons. The methods proposed in this paper can be used to update and maintain the existing CHV. Furthermore, we made some contributions for the cross-language search through annotating MeSH ID for each consumer health term in Chinese.

## Abbreviations

AKA: Also known as
CHT: Chinese consumer health terms
CHV: Consumer Health Vocabularies
CMeSH: Chinese Medical Subject Headings
IAA: Inter-annotator agreement
ICD-10: International Classification of Diseases 10, 10th Edition
NCIt: National Cancer Institute thesaurus)
NLP: Natural Language Processing
SNOMED-CT: Systematized Nomenclature of Medicine-Clinical Terms
UMLS: Unified Medical Language System
